# Synthesis of Highly Crystalline Graphite from Spontaneous Ignition of In Situ Derived Acetylene and Chlorine at Ambient Conditions

**DOI:** 10.3390/molecules25020297

**Published:** 2020-01-11

**Authors:** Nikolaos Chalmpes, Konstantinos Spyrou, Athanasios B. Bourlinos, Dimitrios Moschovas, Apostolos Avgeropoulos, Michael A. Karakassides, Dimitrios Gournis

**Affiliations:** 1 Department of Materials Science & Engineering, University of Ioannina, 45110 Ioannina, Greece; chalmpesnikos@gmail.com (N.C.); konstantinos.spyrou1@gmail.com (K.S.); dmoschov@cc.uoi.gr (D.M.); aavger@uoi.gr (A.A.); mkarakas.cc.uoi@gmail.com (M.A.K.); 2 Physics Department, University of Ioannina, 45110 Ioannina, Greece

**Keywords:** graphite, graphene, calcium carbide, acetylene, bleach, chlorine

## Abstract

We exploited a classic chemistry demonstration experiment based on the reaction of acetylene with chlorine to obtain highly crystalline graphite at ambient conditions. Acetylene and chlorine were generated in-situ by the addition of calcium carbide (CaC_2_) in a concentrated HCl solution, followed by the quick addition of domestic bleach (NaClO). The released gases reacted spontaneously, giving bursts of yellow flame, leaving highly crystalline graphite deposits in the aqueous phase. This was a rather benign alternative towards synthetic graphite, the latter usually being prepared at high temperatures. The synthetic graphite was further utilized to obtain graphene or conductive inks.

## 1. Introduction

Synthetic graphite plays a central role in materials science, displaying numerous real-life applications in the fields of electrolysis, metallurgy, thermal insulation, refractories, brake linings, pencils, heat sinks, conductive inks, batteries, lubricants, neutron moderators, carbon fibers/reinforcement, and graphene production [1,2,3,4]. However, its making often requires high temperatures in order to achieve a high degree of graphitization and crystallinity (ca. 2500 °C) [4]. In this respect, the advancement of new methods towards synthetic graphite under milder conditions is highly recommended.

Calcium carbide (CaC_2_) is considered a useful and inexpensive reagent towards carbon nanomaterials [5,6]. For instance, simple acid hydrolysis of CaC_2_ has resulted in high-quality graphene at ambient conditions [7]. On the other hand, thermal treatment of CaC_2_ with sulfur at 550 °C gave a highly crystalline graphite through a single displacement reaction that is thermodynamically favored at this high temperature [8]. Therefore, CaC_2_ seems to be a potential source of graphitic materials under mild conditions.

A classic reaction used in chemistry demonstrations refers to the reaction of acetylene with chlorine [9]. Both gases are produced in-situ from inexpensive reagents, such as CaC_2_ and domestic bleach (NaClO) in an acidic solution. Once generated, acetylene and chlorine spontaneously ignite to produce bursts of yellow flame and carbon, the latter being identified in this work as a highly crystalline graphite. In other words, we show here that the reaction of acetylene with chlorine spontaneously results in highly crystalline graphite at ambient conditions. We further demonstrate some practical uses of the synthetic graphite in making graphene or conductive inks.

## 2. Results and Discussion

The synthesis of graphite can be summarized by the reactions:

CaC_2_ + 2 HCl→ CaCl_2_ + C_2_H_2_ (1)

NaOCl + 2 HCl → Cl_2_ + NaCl + H_2_O (2)

C_2_H_2_ + Cl_2_ → 2 C_graphite_ + 2 HCl (3)

In Equation (1) calcium carbide releases acetylene by the action of HCl. Simultaneously, the bleach reacts with HCl to liberate chlorine gas, according to Equation (2). Following, the in-situ derived C_2_H_2_ and Cl_2_ react spontaneously as shown in Equation (3) to produce graphite. Based on the standard molar enthalpies and entropies of the products-reactants in Equation (3), it turns out that this is a thermodynamically favored reaction at ambient conditions (298 K, 1 atm: ΔH_rxn_^o^ = −411 kJ mol^−1^, ΔS_rxn_^o^ = −0.039 kJ K^−1^ mol^−1^, ΔG_rxn_^o^ = −400 kJ mol^−1^).

**Figure 1:**
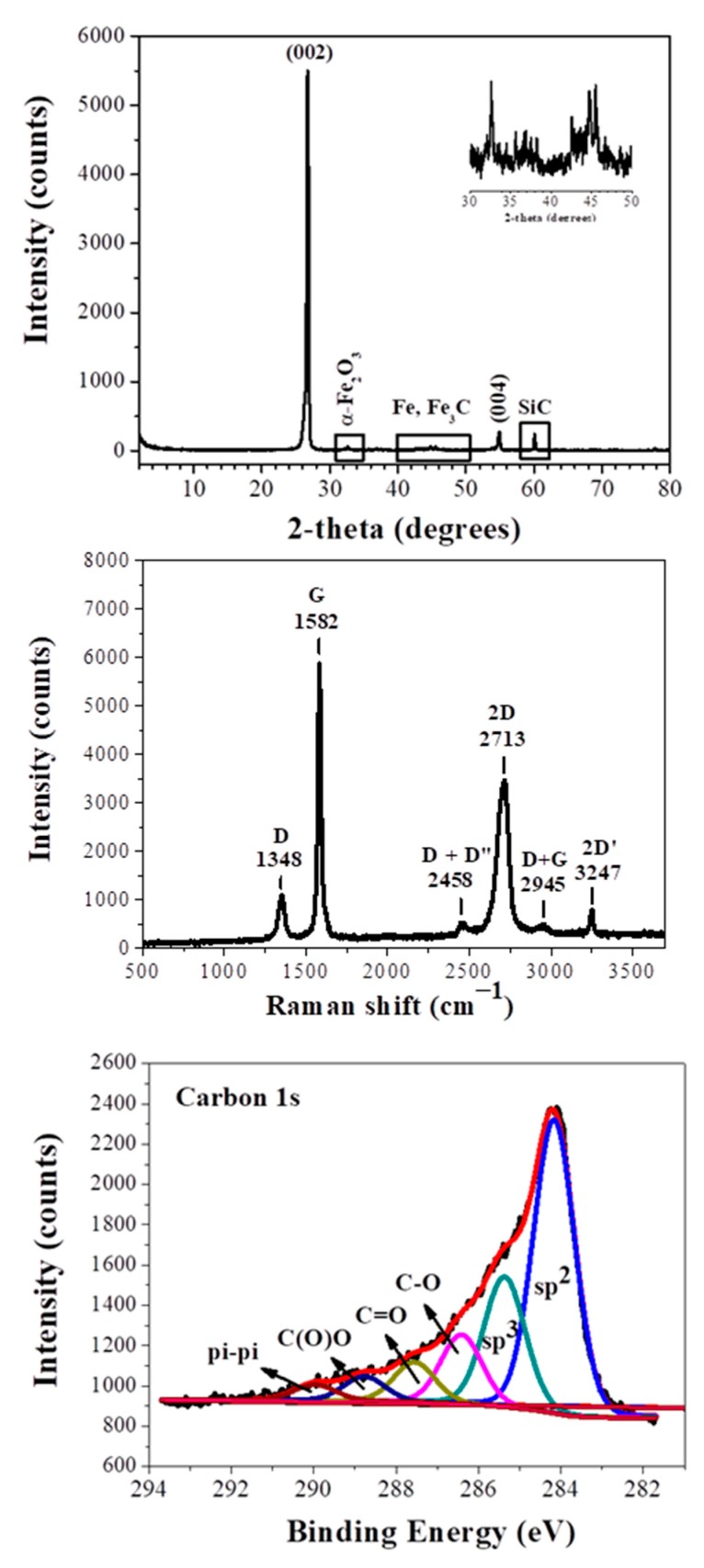
XRD pattern (**top**), Raman spectrum (**middle**), and C1s XPS spectrum (**bottom**) of the synthetic graphite. The top inset shows in magnification the 2-theta region 30–50°.

The XRD pattern (Figure 1, top) of the sample shows a sharp diffraction peak attributed to the (002) reflection of graphite, confirming a high degree of graphitization [10]. The interplanar spacing of this reflection is 3.33Å, very close to that of commercial synthetic graphite (3.34Å) (Alfa Aesar, median 7–10 micron, 99%, Lot: R22A019) (Figure S1, Supplementary Materials). In addition to this peak, we also observe the (004) plane of graphite [10]. Very weak reflections of between 30° and 50° are assigned to iron impurities (α-Fe_2_O_3_, Fe, Fe_3_C) that confer magnetic character to the product, whereas the peak near 60° is assigned to SiC contaminants [6,8]. Such impurities are quite common in CaC_2_-derived carbons and originate from the starting calcium carbide [6,8]. It is also likely that iron impurities may act catalytically in graphite formation [11]. It is worth mentioning that the current synthetic graphite shows a higher X-ray crystallinity than other synthetic graphites recently presented in literature [12].

The synthetic graphite was IR-inactive, but Raman-active [13,14]. The Raman spectrum (Figure 1, middle) displays two strong bands at 1582 cm^−1^ (G: sp^2^ carbon) and 2713 cm^−1^ (2D), as well as weaker bands at 1348 cm^−1^ (D: sp^3^ carbon), 2458 cm^−1^ (D + D”), 2945 cm^−1^ (D + G), and 3247 cm^−1^ (2D’). The peak positions of the 2D and G bands, together with their intensity ratio, are well correlated with multilayer graphite. On the other hand, the symmetric 2D band (e.g., absence of any shoulder on the left side of the band) possibly suggests the additional presence of graphene nanosheets, the latter derived from concurrent acid hydrolysis of CaC_2_ [7]. It should be emphasized that the intensity ratio I_D_/I_G_ of synthetic graphite was found at 0.15, i.e., in the range of 0.1–0.2 expected for highly crystalline graphite. Furthermore, the full-width half-maximum (FWHM) of the sharp G band was 20 cm^−1^, thus additionally indicating the formation of highly crystalline graphite. These features are strikingly superior to those presented elsewhere for synthetic graphite [15]. Most importantly, the Raman spectrum of the sample is quite reminiscent of commercial synthetic graphite (Alfa Aesar, median 7–10 micron, 99%, Lot: R22A019) presented in Figure S2, Supporting Information.

The X-Ray Photoelectron Spectroscopy (XPS) survey of the sample demonstrates carbon and oxygen peaks at percentages of 65.8% and 34.2%, respectively (Figure S3, Supplementary Materials). These values reflect on the surface composition of the thick plates and not on the total carbon content of the sample—the latter is expected to be even higher in the bulk. Τhe high resolution C1s spectrum (Figure 1, bottom) is typical of surface oxidized graphite. Oxidation is expected because of synthesis in open air. The spectrum was deconvoluted into six fitted peaks [16,17]. The more intense peaks, centered at 284.2 eV and 285.4 eV, are attributed to surface sp^2^ and sp^3^ carbon atoms, with sp^2^ carbon being the dominant component. The peak, located at 286.4 eV, is due to C-O groups, while the next one, at a higher binding energy of 287.6 eV, are carbons bound to oxygen in the form C=O. Carboxyl groups were observed at 288.8 eV, whereas a weak peak at very high binding energies (290.0 eV) may be attributed to π–π* transitions.

**Figure 2:**
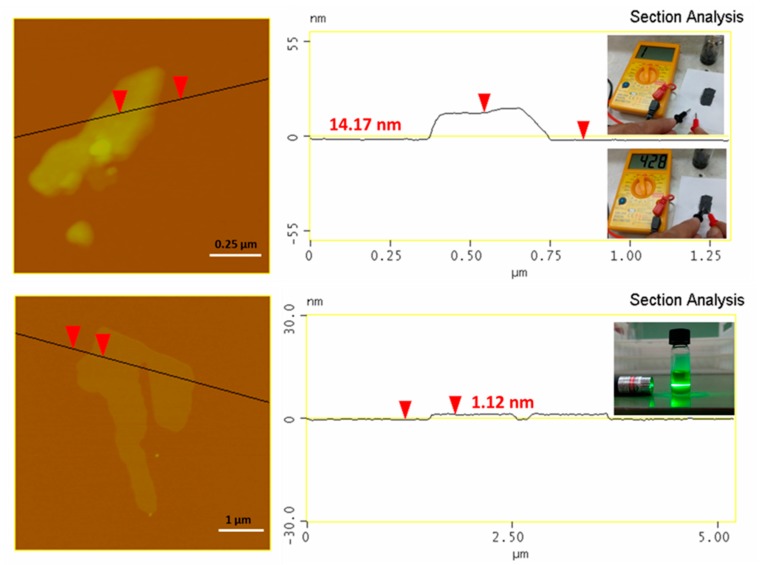
AFM images and height profiles of synthetic graphite (**top**) and derived graphene (**bottom**). The top inset shows the waterborne conductive ink made of water glass and synthetic graphite. The ink is applied on a piece of paper using a small paintbrush, leaving a flexible conductive trace after drying (note that paper itself is an insulator). The bottom inset shows the colloidal dispersion of graphene in DMF exhibiting a strong Tyndall effect.

An Atomic Force Microscopy (AFM) study (Figure 2, top) of the synthetic graphite revealed the formation of graphite plates with a thickness of between 15–20 nm and lateral size in the range of 2–5 μm (e.g., thick micron-sized plates). The thickness of the plates exceeded the value of 5–10 nm for graphene nanosheets, hence pointing to multilayer graphite. In a practical use, we made a conductive ink by simply mixing by hand a small amount of graphite powder with water glass (sodium silicate aqueous solution) as a binder. The ink was spread on a paper, leaving behind a flexible conductive trace after drying (Figure 2, top: inset). In another example, we produced graphene using the liquid-phase exfoliation technique [3]. To this aim, 0.1 g of synthetic graphite was suspended by 3 h under sonication (130 W) in 25 mL of dimethylformamide (DMF) in a sealed glass vial. The suspension was left at rest for three days in order to settle down any solid particulates, and the supernatant was collected as a clear colloid giving a strong Tyndall effect using a green laser pointer (Figure 2, bottom: inset). According to the AFM, the colloid contained micron-sized thin sheets with a thickness of 1–1.3 nm (Figure 2, bottom). Such values are consistent with graphene [18] (graphene ISO standard: ISO/TS 80004-13:2017). A statistical analysis of graphite and graphene thicknesses based on an AFM is included in the supporting information section (Figure S4). Last, for reasons of completeness we have also included some representative TEM images of graphite and graphene as supporting information (Figure S5).

## 3. Materials and Methods

Three grams of CaC_2_ Sigma-Aldrich (St. Louis, MO, USA) technical grade 80%, Lot # STBC8955V) were added at small portions in 20 mL of a concentrated HCl solution (37%) to produce acetylene gas. Every addition of calcium carbide to the HCl solution was accompanied by the quick addition of a small amount of domestic bleach (NaClO). A total of 25 mL bleach was used in the experiment. The bleach and HCl produced chlorine gas that, when combined with acetylene, gave bursts of yellow flames which left a graphite precipitate. The mixture was left at rest for 1 h until no bubbling was observed. The precipitate was rinsed several times with water and acetone prior to drying. Finally, it was treated with a saturated aqueous solution of the chelating agent ethylenediaminetetraacetic acid (EDTA) for 2 h under sonication in order to remove residual calcium compounds. The obtained graphite (yield: 5%) was highly conductive, as tested from large lumps using a two-point multimeter. In addition, the graphite was attracted to a magnet due to iron impurities already present in the calcium carbide. Based on TGA (Figure S7, Supplementary Materials), the impurities accounted for nearly 12% of the sample’s composition. Specifically, iron impurities are very common in synthetic graphite [19]. Furthermore, metallic impurities are sometimes advantageous in electroanalysis, since they provide significantly larger voltammetric currents and enhance electrocatalytic activity [20,21]. Finally, the solid had the lustrous grey appearance of graphite (Figure S6), while its thermal decomposition in air occurred at >600 °C (Figure S7).

Powder X-ray diffraction (XRD) was performed using background-free Si wafers and Cu Ka radiation from a Bruker Advance D8 diffractometer (Bruker, Billerica, MA, USA). Raman spectra were recorded with an RM 1000 Renishaw micro-Raman system using a laser excitation line at 532 nm (Renishaw, Wotton-under-Edge, England). X-ray photoelectron spectroscopy (XPS) measurements were performed in an ultra-high vacuum at a base pressure of 4 × 10^−10^ mbar with a SPECS GmbH spectrometer equipped with a monochromatic Mg Kα source (hv = 1253.6 eV) and a Phoibos-100 hemispherical analyzer. Atomic force microscopy (AFM) images were collected in tapping mode with a Bruker Multimode 3D Nanoscope using a microfabricated silicon cantilever type TAP-300G, with a tip radius of <10 nm and a force constant of approximately 20–75 N m^−1^. Suspensions of the materials with a 0.1% *w*/*v* concentration were prepared and drop-casted onto carbon coated copper grids (CF300-CU-UL, carbon square mesh, CU, 300 mesh from Electron Microscopy Science, Hatfield, England). The drop-casted sections were studied using TEM (JEM HR-2100, JEOL Ltd., Tokyo, Japan) operated at 200 kV in bright-field mode.

## 4. Conclusions

A simple method to prepare highly crystalline graphite through spontaneous ignition of in-situ derived acetylene and chlorine at ambient conditions is reported. The obtained graphite was magnetic due to iron contaminants, and also conductive thanks to a high degree of graphitization. The high crystallinity and morphology of the synthetic graphite were verified by XRD, Raman, XPS, and AFM/TEM techniques. Liquid-phase exfoliation of the solid in DMF produced fine colloidal dispersions of graphene, whereas simple mixing with water glass gave conductive inks. Overall, this is an energy-saving, facile, and inexpensive alternative to manufacturing synthetic graphite at ambient conditions for direct practical uses.

## Supplementary Materials

The following are available online: Figure S1: XRD pattern of the commercial synthetic graphite (Alfa Aesar, median 7–10 micron, 99%, Lot: R22A019), Figure S2: Raman spectrum of the commercial synthetic graphite (Alfa Aesar, median 7–10 micron, 99%, Lot: R22A019), Figure S3: XPS survey of the synthetic graphite, Figure S4: AFM statistical analysis of graphite (a and b images) and graphene (c and d images) for 60 randomly selected nanosheets, Figure S5: TEM of graphene nanosheets (a and b images) and synthetic graphite (c and d images), Figure S6: Grey-lustrous chunks of synthetic graphite, and Figure S7: TGA-DTA trace of synthetic graphite in air.
