# Supplementary material for: Synthesis of Highly Crystalline Graphite from Spontaneous Ignition of In Situ Derived Acetylene and Chlorine at Ambient Conditions
Source: Molecules. 2020 Jan 11;25(2):297. doi: 10.3390/molecules25020297 (PMC7024288; doi:10.3390/molecules25020297)
Supplement: Supplementary file 1 [file molecules-25-00297-s001.pdf]

Supplementary

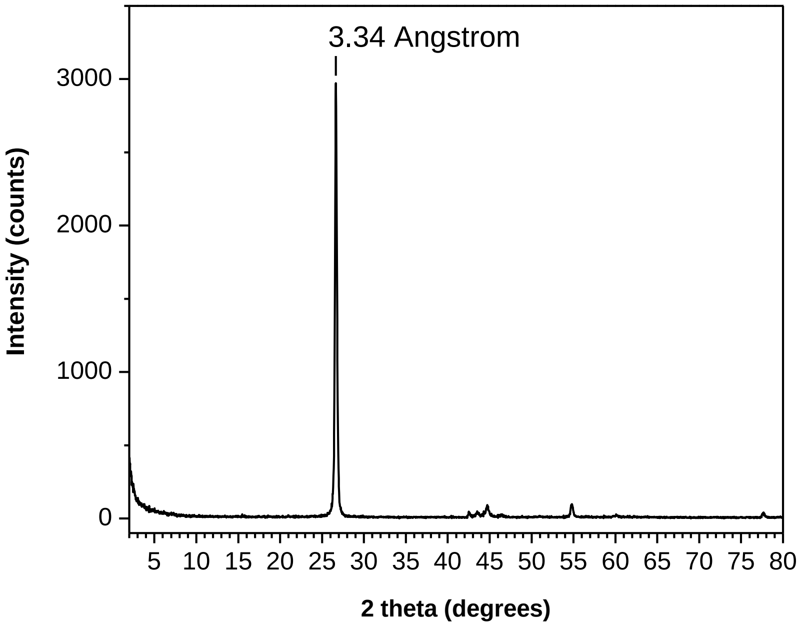

Figure S1. XRD pattern of the commercial synthetic graphite (Alfa Aesar, median 7-10 micron, 99%, Lot: R22A019).

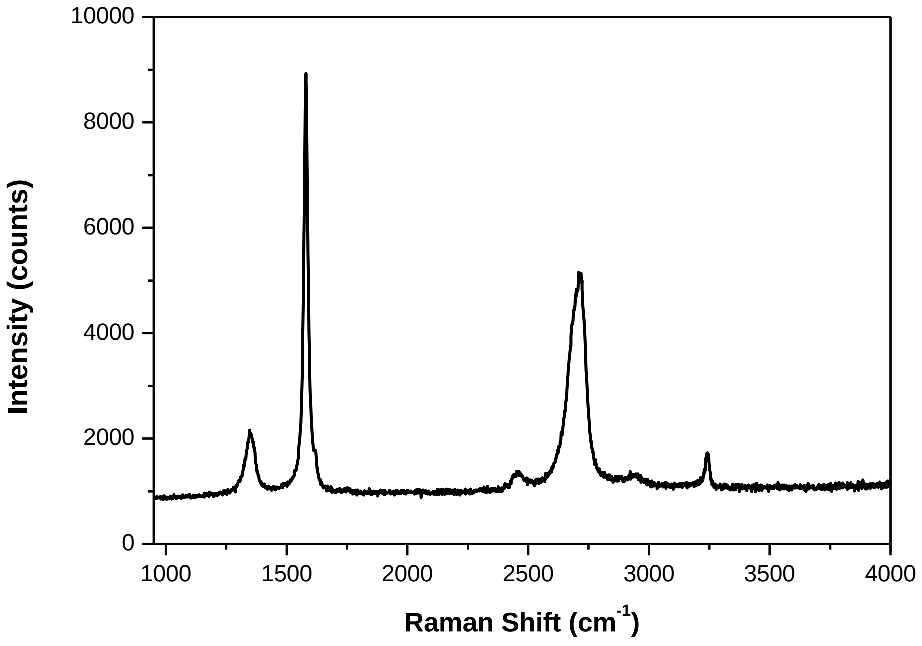

Figure S2. Raman spectrum of the commercial synthetic graphite (Alfa Aesar, median 7-10 micron, 99%, Lot: R22A019).

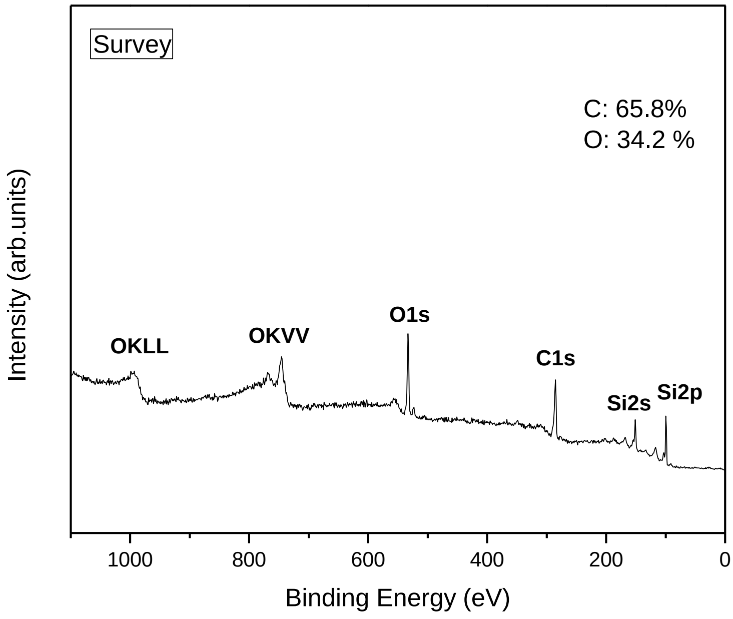

Figure S3. XPS survey of thesynthetic graphite.

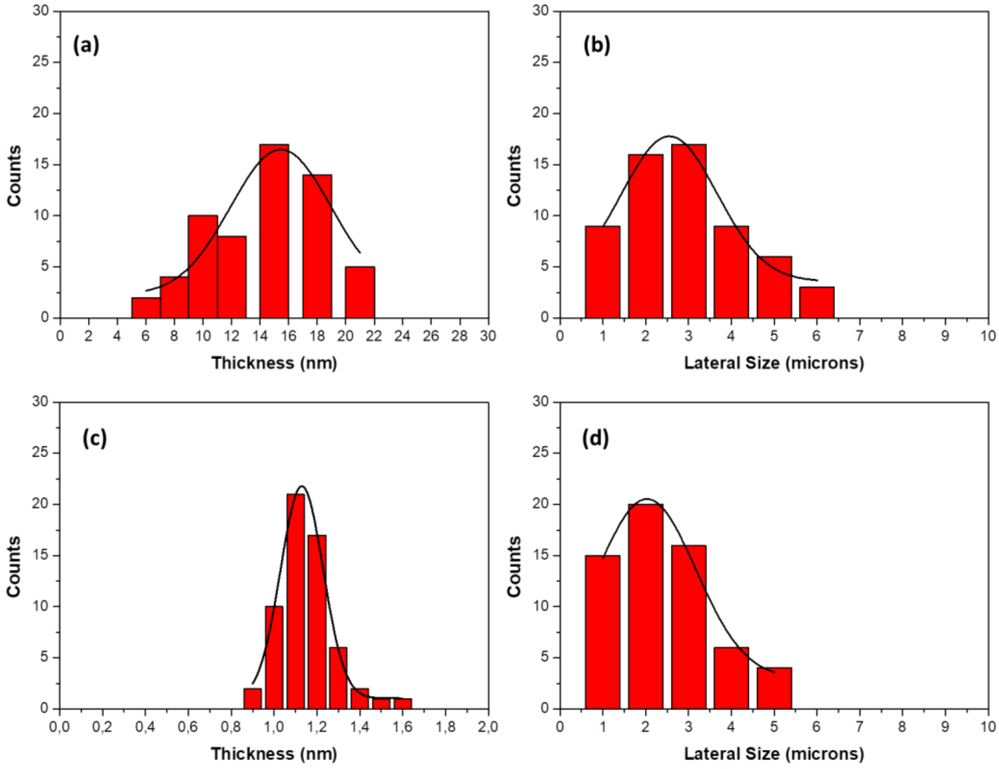

Figure S4. AFM statistical analysis of graphite (a and b images) and graphene(c and d images) for 60 randomly selected nanosheets.

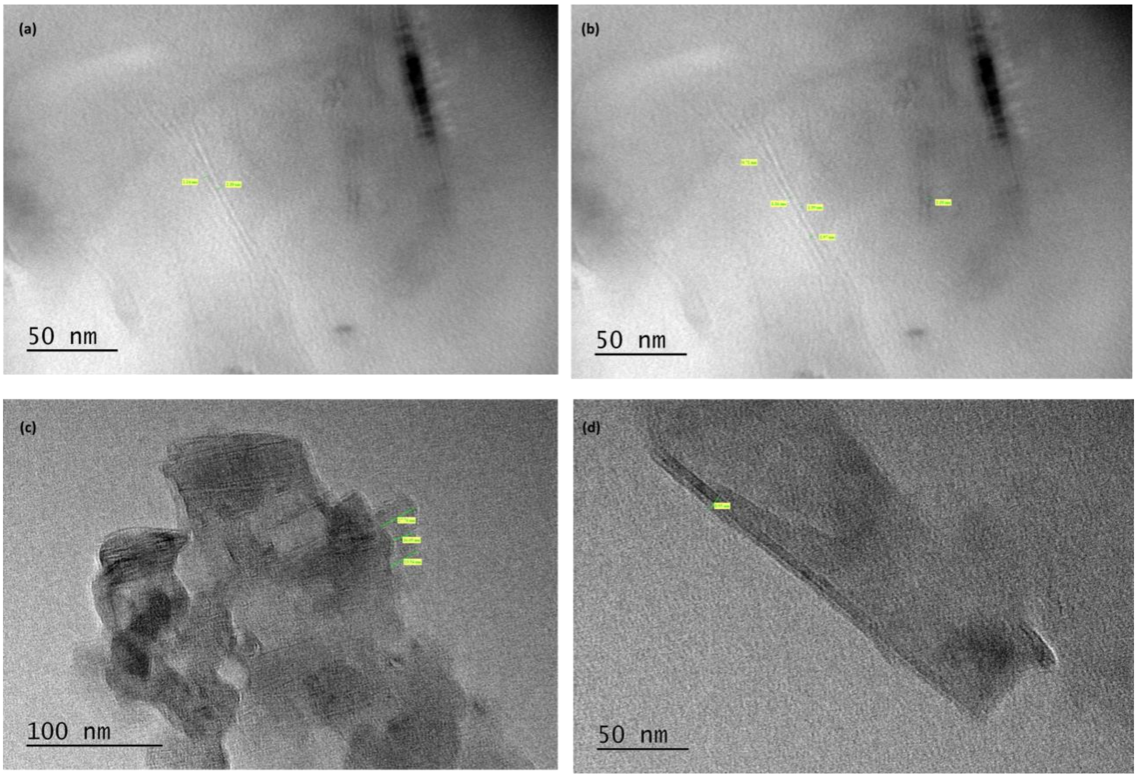

Figure S5. TEM of graphene nanosheets (a and b images) and synthetic graphite (c and d images).

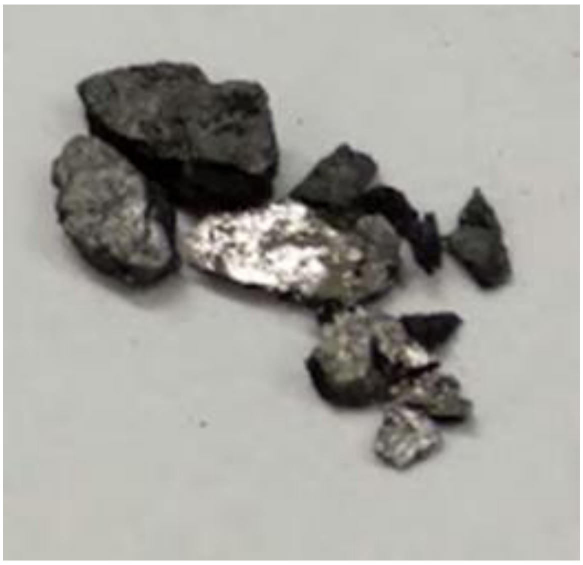

Figure S6. Grey-lustrous chunks of synthetic graphite.

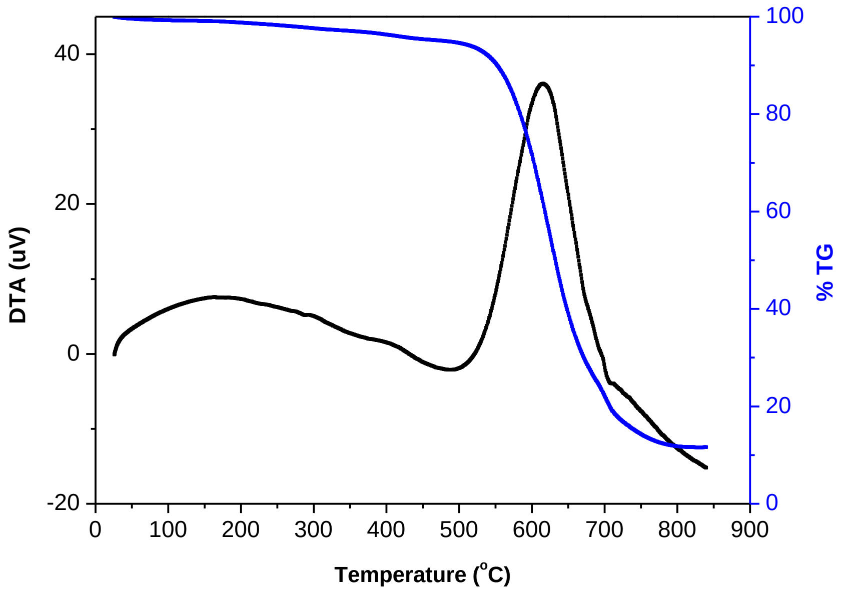

Figure S7. TGA-DTA trace of synthetic graphite in air.
